# Removal of the endoplasma membrane upon sperm cell activation after pollen tube discharge

**DOI:** 10.3389/fpls.2023.1116289

**Published:** 2023-01-26

**Authors:** Naoya Sugi, Rie Izumi, Shun Tomomi, Daichi Susaki, Tetsu Kinoshita, Daisuke Maruyama

**Affiliations:** Kihara Institute for Biological Research, Yokohama City University, Yokohama, Japan

**Keywords:** pollen tube, sperm cell, vegetative nucleus, male germ unit (MGU), double fertilization

## Abstract

In pollen and pollen tubes, immotile sperm cells are enclosed by an inner vegetative plasma membrane (IVPM), a single endomembrane originating from the vegetative-cell plasma membrane. It is widely believed that sperm cells must be removed from the IVPM prior to gamete associations and fusions; however, details of the timing and morphological changes upon IVPM dissociation remain elusive. Here, we report a rapid IVPM breakdown immediately before double fertilization in *Arabidopsis thaliana*. The IVPM was stably observed in coiling pollen tubes when pollen tube discharge was prevented using *lorelei* mutant ovules. In contrast, a semi-*in vivo* fertilization assay in wild-type ovules demonstrated fragmented IVPM around sperm nuclei 1 min after pollen tube discharge. These observations revealed the dynamic alteration of released sperm cells and provided new insights into double fertilization in flowering plants. With a summary of recent findings on IVPM lipid composition, we discussed the possible physiological signals controlling IVPM breakdown.

## 1 Introduction

**Figure 1:**
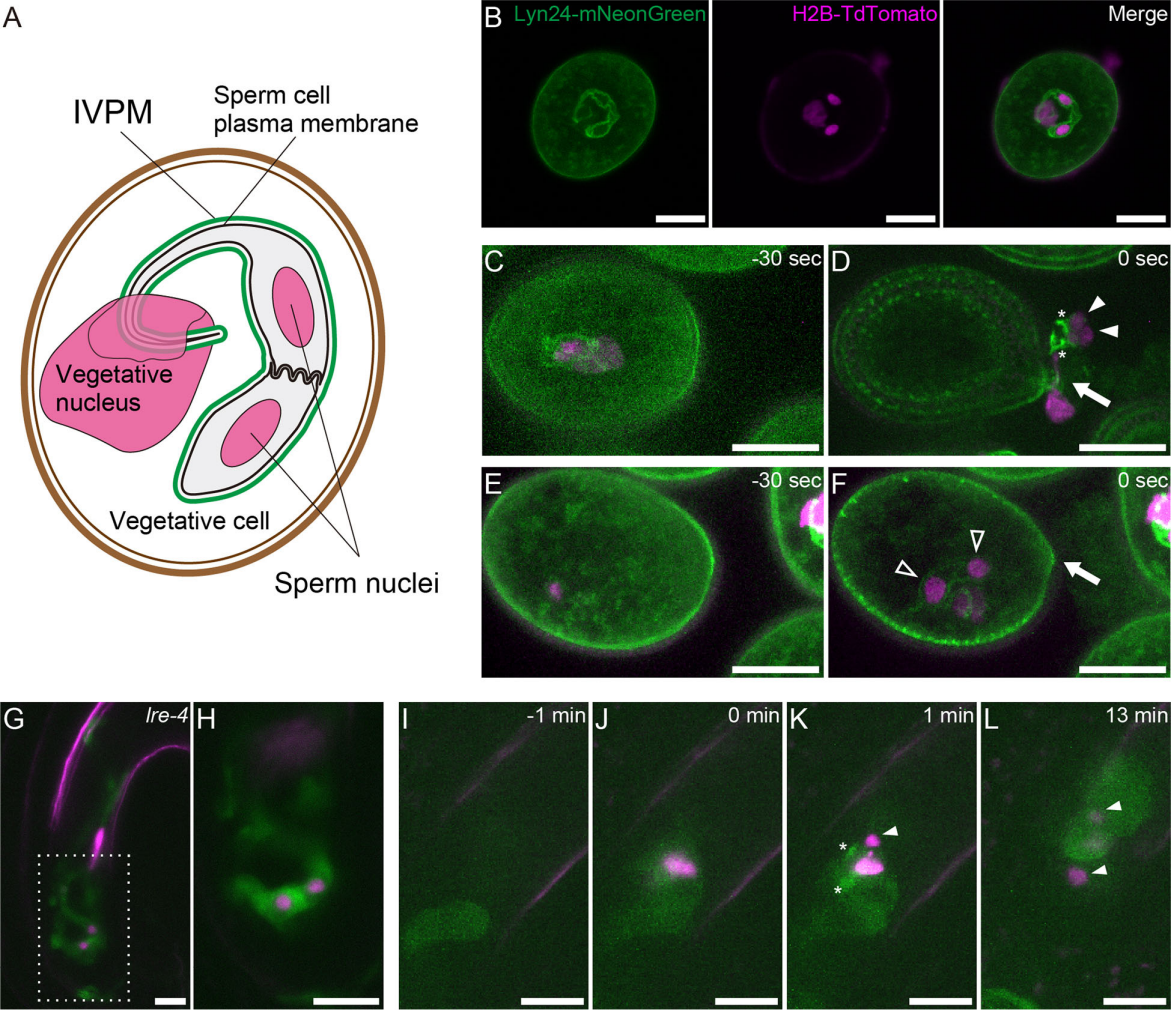
Structure and dynamics of IVPM. **(A)** Schematic drawings of the male germ unit (MGU) in *Arabidopsis* mature pollen. **(B)** Confocal images of a pollen grain in the *pACA3:Lyn24- mNeonGreen; pRPS5A:H2B-tdTomato* double marker. **(C–F)** Maximum intensity projections of *in vitro*-cultured *anx1*/*2* mutant pollen carrying the *pACA3:Lyn24-mNeonGreen; pRPS5A:H2B-tdTomato* double maker. Rapid transition from sperm cell-enclosing IVPM **(C)** to fragmented IVPM **(D)** occurred within 30 sec during sperm cell release. In contrast, sperm cell-enclosing IVPM was persistently observed before **(E)** or after **(F)** pollen tube rupture when the sperm failed to exit the pollen grain. Arrows indicate breaks of germinating pollen tubes; filled arrowheads, sperm nuclei dissociated from IVPM; open arrowheads, sperm nuclei within IVPM; and asterisks, fragmented IVPM. See also **Supplementary Figure 1**. **(G)** Representative projection image of a pollen tube growing in the *lre-4* mutant ovule 12–18 h after pollination. **(H)** Single plane confocal image of the dashed area in **(G)**. **(I–L)** Time-lapse imaging of IVPM breakdown during fertilization. Discharging of the *pACA3:Lyn24-mNeonGreen; pRPS5A:H2B-tdTomato* double marker pollen tube in the wild-type ovule was analyzed using semi-*in vivo* fertilization assay. Arrowheads indicate sperm nuclei. Asterisks indicate fragmented IVPM. See also Supplementary Movie 1. Scale bars: 10 μm.

A rapidly growing pollen tube transports immotile sperm cells to female gametes. Analyzing the dynamic morphological changes of sperm cells is important in understanding the unique reproduction of flowering plants. During male gametogenesis, pollen microspores undergo mitosis twice. The first asymmetric division (pollen mitosis I) produces a vegetative cell and a generative cell. Generative cells are enveloped within vegetative cells. Generative cells undergo symmetrical mitotic division (pollen mitosis II), producing two sperm cells. Following these unusual internalization events, a single membrane derived from the vegetative cell membrane encloses the generative and sperm cells. Electron micrographs of mature pollen demonstrated that the sperm-enclosing endoplasma membrane—inner vegetative plasma membrane (IVPM)—is closely apposed to the plasma membrane in several organisms as symbiotic organelle membranes, such as the inner and outer membranes of mitochondria (Jensen and Fisher, 1970; Russell and Cass, 1981; Dumas et al., 1985). A tail-like protrusion extending from one of the sperm cells tightly connects to the vegetative cell nucleus. This trinuclear assemblage is termed the male germ unit (MGU) (**Figure 1**) (Dumas et al., 1985).

The MGU is maintained in the apical region during pollen tube growth. When the pollen tube reaches the ovule, it discharges the MGU with its cytoplasmic contents deep inside the pistils, where double fertilization occurs: two sperm cells fertilize the egg and central cells. Thus, early studies often used histochemical approaches to investigate reproductive events in pollinated pistils. However, snapshots of fixed samples rarely capture rapid fertilization events, particularly a series of sperm cell behaviors after pollen tube discharge. A detailed time course of double fertilization anticipates the establishment of a semi-*in vivo* fertilization assay in *Arabidopsis thaliana* (Hamamura et al., 2011). Released sperm cells immediately reach the egg cell (8.8 ± 5.5 s), and double fertilization occurs within 7.4 ± 3.3 min. This observation raised new questions about how essential gamete interactions occur in plants within this short period. It has been postulated that IVPM removal and sperm plasma membrane exposure occur immediately after pollen tube discharge to ensure rapid gamete interactions (Sprunck, 2020).

IVPM and sperm plasma membrane are morphologically similar in intact pollen grains and tubes (Li et al., 2013; Billey et al., 2021; Gilles et al., 2021; Motomura et al., 2021). However, it was recently discovered that previously reported IVPM marker proteins are expressed in vegetative cells and exhibit peripheral membrane associations with IVPM. According to the analysis of an IVPM-localized patatin-like phospholipase MATRILINEAL/ZmPHOSPHOLIPASE A1/NOT LIKE DAD (MTL/ZmPLA1/NLD) in maize, IVPM dissociates from sperm cells in both *in vitro* and *in vivo* conditions, such as after osmotic shock-induced pollen tube rupture and in the pollen tube receiving ovules, respectively (Gilles et al., 2021). However, the timing and morphological changes of IVPM breakdown remain elusive. We report the observation of rapid IVPM breakdown after pollen tube discharge, the mechanisms underlying IVPM stability, trigger signals of IVPM breakdown, and future directions of cell biology focusing on IVPM.

## 2 Material and methods

### 2.1 Plant materials and growth conditions

*Arabidopsis thaliana* Columbia-0 was used as the wild-type (WT) plant. The *pRPS5A:H2B-tdTomato pACA3:Lyn24-mNeonGreen* double marker has been previously described (Motomura et al., 2021). The *lorelei-4* (*lre-4*) mutant (SAIL_8_D07) was provided by Dr. Palanivelu (Univ. Arizona) (Tsukamoto et al., 2010). The double mutant of *anxur1-1* (*anx1-1*) and *anx2-2* was provided by Dr. Miyazaki through Dr. Mizuta (Nagoya Univ.) (*anx1-1*: SALK_016179, *anx2-2*: SALK_133057). Seeds were germinated on Murashige-Skoog medium and transferred to soil. Plants were grown at 22°C under continuous lighting conditions.

### 2.2 Microscopy

Mature pollen grains and *lre-4* ovules were mounted in 5% sucrose solution. Samples were observed using a confocal laser-scanning microscope equipped with a 63× objective lens (Leica TCS SP8; Leica, Wetzlar, Germany). The mNeonGreen was excited at 506 nm laser irradiation of the white light laser, and the fluorescence was detected using a Hybrid Detector (HyD) with a range of 511–553 nm. The tdTomato was excited at 554 or 558 nm laser irradiation of the white light laser, and the fluorescence was detected using an HyD with a range of 559–670 nm. Time-lapse images of the semi-*in vivo* fertilization assay was acquired every minute using the G/R split mode of an inverted microscope (IX-73; Olympus, Tokyo, Japan) equipped with a 60× objective lens, a spinning disk confocal scanning unit (CSU-W1; Yokogawa, Tokyo, Japan), and an sCMOS camera (Zyla 4.2; Andor, Belfast, Northern Ireland).

### 2.3 Observation of *in vitro* pollen tube rupture

The pollen grains were placed on pollen tube growth medium (0.01% boric acid, 5 mM CaCl_2_, 5 mM KCl, 1 mM MgSO_4_, 10% sucrose, 10 μM epibrassinolide, 1.5% NuSieve GTG agarose, pH7.5) solidified on a glass bottom dish (D11130H; Matsunami Glass, Osaka, Japan). Pollen tube rupture was observed using the spinning disk confocal microscopy during incubation at 22°C.

### 2.4 Semi-*in vivo* fertilization assay

Semi-*in vivo* fertilization assays were performed as previously described with minor modifications (Susaki et al., 2017). Emasculated WT pistils were cut beneath the style using a 27-gauged needle. The pistils were placed on pollen tube growth medium (0.001% boric acid, 1.27 mM Ca (NO_3_)_2_, 0.4 mM MgSO_4_, 14% sucrose, 10 μM epibrassinolide, 1.5% NuSieve GTG agarose, pH7.0) solidified on a glass bottom dish (D11130H; Matsunami Glass, Osaka, Japan). Afterward, the stigma was pollinated with pollen from the *pRPS5A:H2B-tdTomato pACA3:Lyn24-mNeonGreen* plant. WT ovules were dissected from the emasculated pistils and aligned in front of the pollinated style. After 3 h of incubation at 22°C, pollen tubes reaching the ovules were observed using spinning disk confocal microscopy.

## 3 Results

To visualize IVPM dynamics, we used *pACA3:Lyn24-mNeonGreen*; *pRPS5A:H2B-tdTomato* double marker line (Motomura et al., 2021). The *pRPS5A:H2B-tdTomato* is a ubiquitous nuclear marker (Maruyama et al., 2013). Lyn24 is an N-terminal 24 amino acid of the murine Lyn protein that employs myristoylation and palmitoylation signals. The Lyn24-mNeonGreen fusion protein expressed from the vegetative cell–specific *ACA3* promoter efficiently labels the IVPM (Li et al., 2013). Confocal images of mature pollen grains showed eyeglass-shaped IVPM encasing two sperm nuclei and a tail structure connected to the vegetative nucleus (**Figure 1**). To observe the dynamics of IVPM during pollen tube discharge, we first performed *in vitro* assay of the *anx1-1 anx2-2* mutant (*anx1*/*2*) harboring *pACA3:Lyn24-mNeonGreen pRPS5A:H2B-tdTomato* markers with 30-s intervals. ANX1 and ANX2 are *Catharanthus roseus* receptor-like kinase 1-like (CrRLK1L)-type receptors that regulate the integrity of growing pollen tubes (Boisson-Dernier et al., 2009; Miyazaki et al., 2009). The *anx1*/*2* mutant pollen tubes ruptured soon after germination on the medium, which is expected to mimic the physiological state of native pollen tube reception. Among 13 cases of spontaneous discharge, nine showed immediate IVPM fragmentation and sperm cell separation after the release of MGUs from pollen grains (**Figures 1C**, **Supplementary Figure 1A**). In the other four cases, however, MGUs remained in the pollen and IVPM fragmentation was not observed (**Figures 1E**, **Supplementary Figure 1B**). These *in vitro* observations suggest that IVPM integrity is maintained until complete sperm cell release, and IVPM breakdown occurs through rapid IVPM fragmentation.

To examine IVPM in ovules prior to pollen tube reception, we analyzed *lre-4* mutant pistils pollinated with the double marker pollen. LORELEI is a GPI-anchored protein expressed in synergid cells and regulates pollen tube reception (Capron et al., 2008; Tsukamoto et al., 2010). As reported previously, the pollen tube exhibited overgrowth in the synergid cells of *lre-4* mutant ovules 12–18 h after pollination (**Figures 1G**). Notably, we observed an eyeglass-shaped IVPM pattern in coiling pollen tubes (n = 21), confirming a stable IVPM just before pollen tube discharge.

We then performed a semi-*in vivo* fertilization assay to monitor native IVPM breakdown in ovules. In this assay, WT ovules and pistils pollinated with the double marker pollen were incubated together on a medium, and the pollen tube discharge and double fertilization were captured using time-lapse imaging with 1-minute intervals (**Figures 1I**). Among 13 ovules analyzed, mNeonGreen signal of intact IVPM was detected around sperm nuclei before pollen tube discharge in five ovules (**Supplementary Movie 1**ovule #2 and #3). However, the IVPM signal became fragmented or ambiguous within one min after pollen tube rupture, suggesting that rapid IVPM breakdown coincided with sperm cell release (**Figures 1J**, **Supplementary Movie 1**ovule #1, #2). Thereafter, two sperm nuclei migrated in different directions, indicating a decisive feature of plasmogamy that showed the beginning of the nuclear congression stage in the egg and central cells (**Figure 1**). Overall, we observed a dynamic transition in IVPM stability upon pollen tube discharge.

## 4 Discussion

### 4.1 Pollen tube discharge triggers rapid transition in IVPM stability

IVPM is supposed to dissociate from sperm cells to allow quick gamete associations after discharge; although, membrane dynamics remain elusive (Sprunck, 2020). Through *in vitro* and semi-*in vivo* assays, we observed immediate IVPM fragmentation after sperm cell release. IVPM breakdown displayed good contrast to IVPM robustness before pollen tube discharge. These observations highlighted the rapid transition in IVPM stability.

WT pollen tubes maintained an eyeglass-like IVPM morphology throughout the growth phase (**Figures 1G**). In the pollen tube, the MGU is continuously exposed to mechanical stresses: stretching, twisting, and moving back and forth in intense protoplasmic flow (Schattner et al., 2020). The MGU structure, particularly the tail-like connection, becomes fragile after reducing cellular stiffness by disrupting the coordination of motility between a pair of sperm cells and the vegetative nucleus (Zhou and Meier, 2014; Motomura et al., 2021). We assume that IVPM has sophisticated homeostasis systems that maintain membrane integrity to resist excess mechanical forces and spontaneous membrane breaks. For example, the ESCRT-III complex is a good candidate for controlling IVPM integrity because it promotes membrane repair in a variety of organelles (Gatta and Carlton, 2019; Isono, 2021). The coordination of lipid biosynthesis and proper membrane trafficking would also contribute to IVPM integrity by maintaining the tight membrane association between IVPM and sperm plasma membrane.

**Figure 2:**
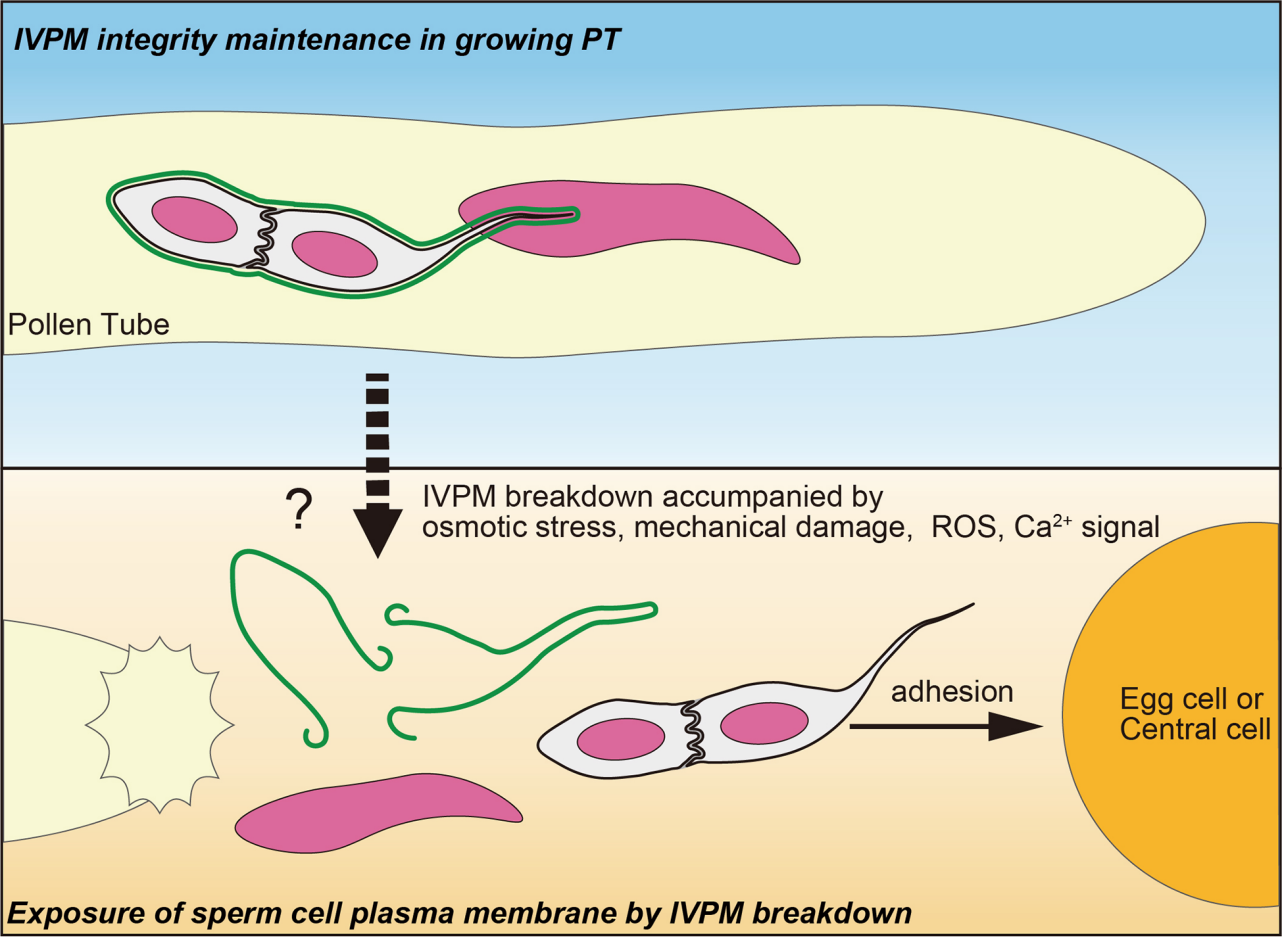
A fine control of IVPM breakdown prior to gamete interaction. Upper panel indicates stable IVPM whose integrity is maintained until pollen tube (PT) discharge. Lower panel shows selective elimination of IVPM after PT discharge. Trigger signal(s) of the IVPM breakdown would arise simultaneously with PT discharge, and thus may be caused by osmotic stress, mechanical damage, ROS and/or Ca^2+^ signal. Due to IVPM breakdown, sperm cells must expose their plasma membranes beforehand to adhere, recognize, and fuse with the egg or central cells.

Released sperm cells immediately showed IVPM fragmentation and separated from each other (**Figures 1C, D**). Such a finding was reported in earlier studies, where sperm cells were isolated from mature pollen grains or pollen tubes (Russell, 1986; Zhang et al., 1992; Borges et al., 2008; Russell et al., 2017). It is still unclear whether these *in vitro* sperm cell separations reflect native IVPM breakdown because mechanistic damage by homogenization or osmotic shock could induce artificial IVPM removal during the isolation procedures. By contrast, we directly observed IVPM breakdown using systems closer to the *in vivo* conditions, which clarified the timing of IVPM breakdown unanswered by the analyses of pollinated pistil in *Plumbago* and maize (Russell, 1983; Gilles et al., 2021). According to the original study of *Arabidopsis* imaging of double fertilization, gamete mergers occur within 7.4 ± 3.3 min after pollen tube discharge (Hamamura et al., 2011). During a short period, sperm cells must complete various events, including fine adjustment of sperm cell position (Huang et al., 2015) and consecutive interactions of male and female gametes, including adhesion, recognition, and fusion (Igawa et al., 2013; Sprunck, 2020). Furthermore, recent studies have depicted a scenario of sperm cell activation prior to gamete fusion, in which egg cell-secreted EC1 family peptides trigger the relocation of the *bona fide* fusogenic factor GCS1-HAP2 on the surface of the sperm plasma membrane in a tetra-spanning membrane proteins DMP8 and DMP9 dependent manner (Sprunck et al., 2012; Cyprys et al., 2019; Wang et al., 2022). Rapid IVPM breakdown could be the initial step in sperm cell activation, which exposes the sperm cell surface and allows EC1-mediated activation and subsequent gamete interactions. Thus, analyzing the two opposing states of IVPM (**Figure 2**)—stable and vulnerable—and understanding the transition mechanisms of IVPM stability will become important issues for future research on double fertilization.

### 4.2 Possible trigger signal of IVPM breakdown

Plants must control the timing of IVPM breakdown because even a slight delay or precocious IVPM disappearance from sperm cells could compromise double fertilization and reduce reproductive fitness. Abiotic stimuli upon pollen tube discharge, including osmotic shock or abrasion on cell wall surface, may explain such a timing control. Intact sperm cell pairs were obtained from various species by employing moderate isolation protocols; some studies detected IVPM capsule (Taylor et al., 1991; Southworth and Kwiatkowski, 1996; Sprunck et al., 2012; Gilles et al., 2021). IVPM behavior in *anx1/2* mutant was also consistent with the abiotic stimulation hypothesis (**Figures 1C**). However, these *ex vivo* releases would provide non-native physiological conditions to the sperm cells. Moreover, this hypothesis does not explain well the distinctive selectivity of IVPM breakdown discussed below. Hence, current data cannot exclude the relevance of other signaling cues at peak during intimate male–female interaction.

Putative signals that trigger IVPM breakdown should be temporarily elevated at the time of pollen tube rupture. Pollen tube cell wall integrity is regulated using an autocrine system, in which CrRLK1L-type receptors, ANX1, ANX2, BUDDHA’S PAPER SEAL 1 (BUPS1), and BUPS2 receive RAPID ALKALINIZATION FACTOR 4 (RALF4) and RALF19 peptides at the pollen tube tips (Boisson-Dernier et al., 2009; Miyazaki et al., 2009; Ge et al., 2017; Mecchia et al., 2017; Zhou et al., 2021). A competitive RALF34 ligand expressed in the ovules disrupts cell wall integrity before tube discharge. However, there is little evidence that the maximum signal of RALF34 can accurately determine the timing of IVPM breakdown *via* the ANX/BUPS pathway. Because *anx1/2* mutant did not show IVPM fragmentation before MGU release, the ANX/BUPS pathway unlikely determines the timing of IVPM breakdown (**Figures 1C**, **Supplementary Figure 1**).

Inorganic environmental changes surrounding the MGU could possibly trigger IVPM breakdown. Reactive oxygen species (ROS), most likely nitric oxide, are produced at high concentrations in synergid cells; *lre* mutants exhibit lower ROS levels than that of the WT (Duan et al., 2014; Duan et al., 2020). The characteristic pollen tube overgrowth phenotype in *lre* mutant ovules can be phenocopied *via* scavenger treatment in WT pistils, suggesting an important role of ROS in pollen tube rupture (Duan et al., 2014). However, these observations are insufficient to predict the ROS-dependent IVPM breakdown mechanism, as dynamic changes in ROS levels during fertilization remain to be analyzed. If synergid cells exhibited a clear oxidative burst upon pollen tube reception, highly membrane-permeable ROS would be an ideal intercellular signal controlling the delicate spatiotemporal activation of sperm cells and/or IVPM breakdown.

Compared with other signaling molecules, the dynamics of signal change were best characterized in cytoplasmic Ca^2+^ during double fertilization. In the time-lapse imaging of semi-*in vivo* fertilization assay using genetically encoded Ca^2+^ sensors, two synergid cells began to show Ca^2+^ oscillation immediately after pollen tube arrival (Ngo et al., 2014). The synergid cells and pollen tube produce a steep cytoplasmic Ca^2+^ elevation upon pollen tube rupture (Iwano et al., 2012; Ngo et al., 2014). Moreover, Ca^2+^ is considered as a putative gamete activator because Ca^2+^ spikes were also observed in the egg and central cells upon pollen tube discharge (Denninger et al., 2014; Hamamura et al., 2014). However, the inhibition of Ca^2+^ influx severely affects pollen tube growth and discharge (Duan et al., 2014). Simple pharmacological and genetic approaches are not applicable to semi-*in vivo* fertilization assays. Thus, decoding the physiological role of Ca^2+^ spikes in IVPM breakdown would be challenging without considerable technical advances in the future.

### 4.3 Molecules required for selective IVPM elimination

One of the most striking features of IVPM breakdown is its high membrane selectivity: IVPM breakdown must occur without damaging the sperm plasma membrane closely apposed with IVPM until pollen tube rupture. This selectivity may be because of its unique phospholipid composition: enriched phosphatidylserine and phosphatidylinositol 4,5-bisphosphate (Gilles et al., 2021). Anionic phospholipids are also enriched in the tips of pollen tube plasma membrane and play an important role in pollen tube growth (Helling et al., 2006; Zhou et al., 2020). It is interesting if membrane-degenerating factors are recruited to the anionic phospholipid–rich membrane domains and induce selective and simultaneous membrane rupture at the pollen tube tip and IVPM. Among the limited number of IVPM proteins reported recently, Rho of Plant (ROP) proteins and MTL/ZmPLA1/NLD exhibit peripheral membrane localization *via* post-translational protein modifications such as palmitoylation or myristoylation (Li et al., 2013; Gilles et al., 2021). MTL/ZmPLA1/NLD also contains positively charged amino acids around the acyl modification motif, which reinforce the association with negatively charged phospholipids in IVPM. Understanding the basis of the protein-localization mechanism of IVPM would be an important clue to narrowing down the candidates for putative membrane-degenerating factors.

### 4.4 Future direction for the study of IVPM breakdown and double fertilization

s the correct subcellular localization of the MTL/ZmPLA1/NLD, an agronomically important haploid inducer protein, has been identified as IVPM, the unique sperm-enclosing membrane has come into the spotlight (Gilles et al., 2021). Although haploid induction in the *mtl*/*Zmpla1*/*nld* mutant is likely to be caused by sperm cell damage due to elevated ROS production, the importance of rapid and selective elimination of IVPM is evident in sexual reproduction of flowering plants (Jiang et al., 2022). Our current knowledge of the unique phospholipid composition and specifically localized proteins is indispensable for developing a novel experimental system to analyze IVPM breakdown. For example, IVPM markers are necessary to establish an efficient fractionation protocol to isolate intact IVPM with the entire MGU structure. Intact MGU can be used in an *in vitro* assay for IVPM breakdown and identification of novel IVPM-specific proteins through mass spectrometry analysis. This progress will lead to more substantial studies on the selective IVPM breakdown mechanism and its triggering signal that culminates in pollen tube rupture.

## Data availability statement

The original contributions presented in the study are included in the article/**Supplementary Material**. Further inquiries can be directed to the corresponding author.

## Author contributions

DM designed the study, conducted the time-lapse imaging. NS observed *anx1*/*2* mutant. RI, DS, and DM observed *lorelei* mutant. ST captured confocal images of pollen grains. NS and DM wrote the manuscript. TK provided critical advice and reviewed the manuscript. All authors contributed to the article and approved the submitted version.
