# Supplementary material for: Removal of the endoplasma membrane upon sperm cell activation after pollen tube discharge
Source: Front Plant Sci. 2023 Jan 26;14:1116289. doi: 10.3389/fpls.2023.1116289 (PMC9909283; doi:10.3389/fpls.2023.1116289)
Supplement: Supplementary file 1 [file DataSheet_1.pdf]

Supplementary Material

Removal of the endoplasma membrane upon sperm cell activation after pollen tube discharge

Naoya Sugi, Rie Izumi, Shun Tomomi, Daichi Susaki, Tetsu Kinoshita, Daisuke Maruyama*

*** Correspondence:** Daisuke Maruyama: dmaru@yokohama-cu.ac.jp

Supplementary Movie Legend

Supplementary Movie 1. IVPM breakdown during fertilization.

A time-lapse movie of a semi-*in vivo* fertilization assay that captured discharges of *pACA3:Lyn24-mNeonGreen; pRPS5A:H2B-tdTomato* double marker pollen tubes in wild-type ovules at 1 min intervals. In ovule #1, fragmented IVPM was clearly detected around released sperm nuclei (corresponding to Figure 1I–L). In ovule #2, the transition from stable to fragmented IVPM was observed within 1 min during pollen tube discharge. In ovule #3, IVPM fragmentation was less evident but the sperm cell-surrounding IVPM pattern could be clearly distinguished immediately before pollen tube discharge. Time stamp: the last frame just after the rapid movement of sperm cells was designated at the moment of the pollen tube discharge, i.e., 0 min. Filled arrowheads indicate sperm nuclei dissociated from IVPM; open arrowheads, sperm nuclei within IVPM; and asterisks, fragmented IVPM. The # symbol indicates IVPM before pollen tube discharge.

Supplementary Figure

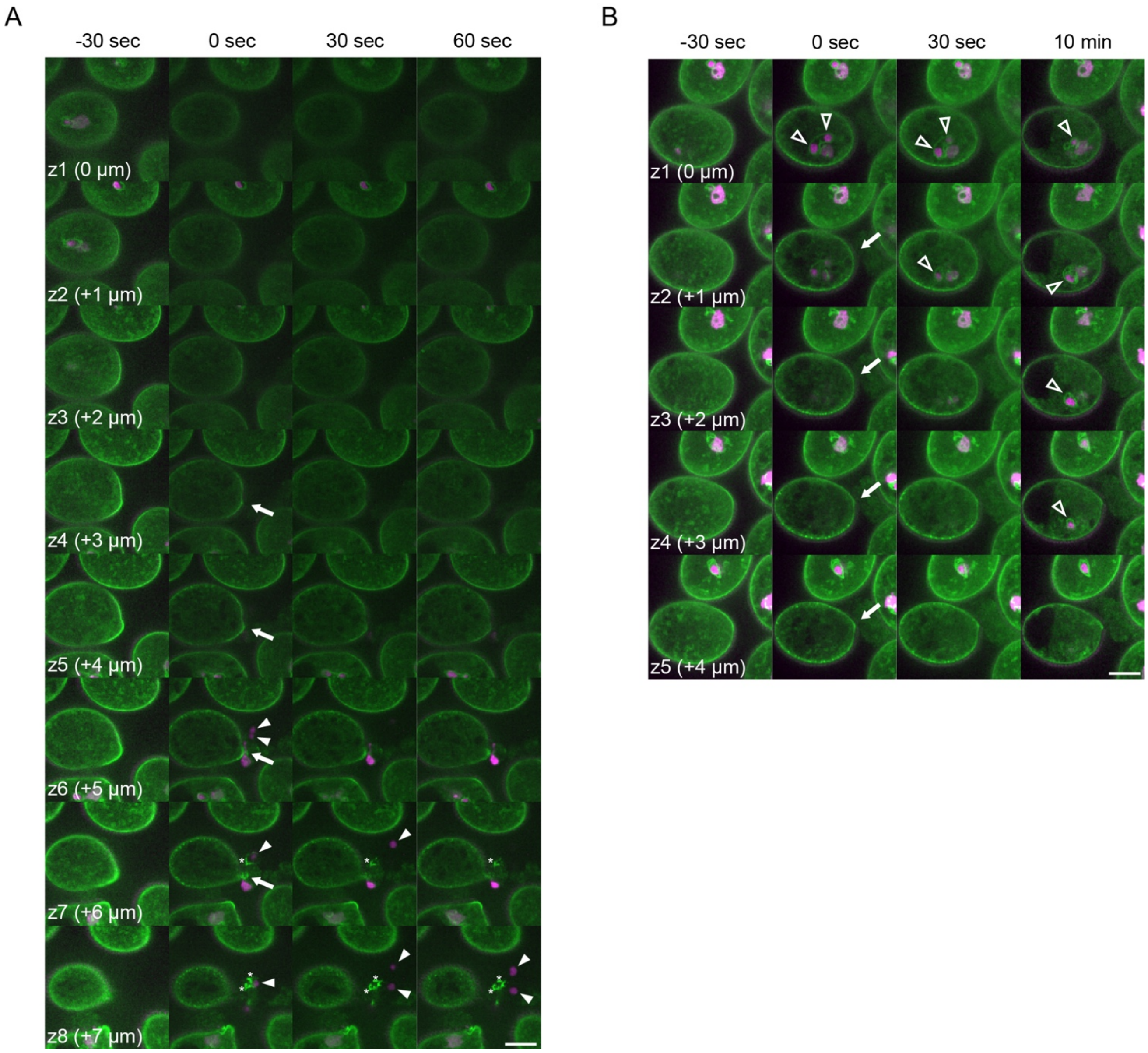

Supplementary Figure 1. Montaged confocal images of *in vitro*-cultured *anx1 anx2* mutant pollen carrying the *pACA3:Lyn24-mNeonGreen; pRPS5A:H2B-tdTomato*.

(A) Rapid fragmentation and removal of IVPM observed after sperm cell release. **(B)** Eyeglass-shaped IVPM observed even after the spontaneous pollen tube discharge. Note that stable IVPM were only found in pollen grains that failed to release sperm cells. Arrows indicate breaks of germinating pollen tubes. Filled arrowhead indicates sperm nuclei dissociated from IVPM; arrowhead, sperm nuclei within IVPM; and asterisks, fragmented IVPM. Scale bars: 10 µm.
